# The transcription elongation factor TCEA3 promotes the activity of the myogenic regulatory factors

**DOI:** 10.1371/journal.pone.0217680

**Published:** 2019-06-03

**Authors:** Noor Kazim, Abhinav Adhikari, Judith Davie

**Affiliations:** Department of Biochemistry and Molecular Biology and Simmons Cancer Institute, Southern Illinois University School of Medicine, Carbondale, Illinois, United States of America; University of Minnesota Medical School, UNITED STATES

## Abstract

The transcription elongation factor TFIIS is encoded by a three member gene family in vertebrates. Here we show that one member of this family, TCEA3, is upregulated during skeletal muscle differentiation and acts to promote gene activation by the myogenic regulatory family of transcription factors, which includes MyoD and myogenin. We show that myogenin is a direct regulator of *Tcea3*. Myogenin binds to the *Tcea3* promoter and is required to recruit RNA polymerase II. TCEA3 can bind to both myogenin and MyoD and is co-recruited with the MRFs to promoters dependent on the MRFs. Depletion of myogenin inhibits the recruitment of TCEA3, suggesting that the interaction of TCEA3 with the MRFs serves to aid in recruitment to target promoters. Like TFIIS, we show that TCEA3 interacts with RNA polymerase II. TCEA3 travels with the elongating RNA polymerase II in the coding region of genes and depletions of TCEA3 inhibit the recruitment of RNA polymerase II to promoters. In proliferating cells, TCEA3 expressed at low levels and is present in both the nucleus and cytoplasm. However, upon differentiation, TCEA3 is upregulated and transported exclusively to the nucleus. Thus, our data show that TCEA3 is a required co-factor for MRF driven gene expression during myogenesis.

## Introduction

TFIIS is a transcription elongation factor conserved through the eukaryotic linage. In vertebrates; including frog, mouse and human, TFIIS is represented by a gene family which includes *TCEA1*, *TCEA2* and *TCEA3* [1]. *TCEA1* is the gene most closely related to the sole TFIIS present in non-vertebrates and best characterized in the yeast, *S*. *cerevisiae* [2]. TCEA1 is ubiquitously expressed and has been shown to regulate the proliferation and differentiation of myeloid cells [3]. Recent work suggest that TCEA1 can also promote proliferation in hepatic carcinoma cells [4]. However, the functions of the other TFIIS isoforms in normal cells or cancer cells are poorly defined. TCEA3 expression is tissue restricted and is known to be expressed in intestine, heart, testis, kidney and skeletal muscle [1]. TCEA3 is also expressed in mouse embryonic stem cells and functions in lineage determination though regulation of the Nodal pathway [5]. TCEA3 has also been shown to regulate the pluripotent differentiation potential of mouse embryonic stem cells [6]. TCEA3 has also been shown to function as a tumor suppressor in ovarian cancer cells [7] and promotes apoptosis in gastric cancer cells [8]. TCEA3 has recently been shown to promote the differentiation of bovine skeletal muscle cells [9].

The regulation of skeletal muscle precursor cell proliferation and subsequent fusion to form myotubes is controlled by a group of highly related transcription factors known as the Myogenic Regulatory Factors (MRFs) [10]. The MRFs, which include Myf5 (*Myf5*), MyoD (*Myod1*), Mrf4 (*Myf6*) and myogenin (*Myog*), are basic helix-loop-helix (bHLH) transcription factors that are required for myogenesis [11].

In this study, we found that *Tcea3* is directly regulated by MYOG. The upregulation of TCEA3 upon differentiation is also correlated with a cytoplasmic to nuclear translocation of TCEA3. TCEA3 binds to both MYOG and MYOD1 and promotes the activities of the MRFs to activate MRF driven gene expression. TCEA3 binds to RNAPII and travels with elongating RNAPII to promote the transcription elongation of muscle specific genes.

## Material and methods

### Cell culture

Proliferating C2C12 myoblasts (ATCC) were grown in Dulbecco’s modified Eagle medium (DMEM) (Hyclone, Thermo Scientific, Waltham MA) supplemented with 10% fetal bovine serum (Hyclone). To induce differentiation into myotubes, C2C12 cells were grown to 70% confluence and then transferred to differentiation medium (DMEM supplemented with 2% horse serum) (Hyclone, Thermo Scientific, Walthman MA), 10T1/2 cells (ATCC) and HEK293 (ATCC) cells were grown in DMEM supplemented with 10% fetal bovine serum.

### Cloning

TCEA3 was PCR amplified from cDNA reverse transcribed from RNA isolated from C2C12 cells differentiated for four days. The PCR amplified fragments were cloned into the pEF6/V5 His TOPO TA expression vector and clones were confirmed by sequencing.

### Quantitative real time PCR

RNA extraction from cells was done using Trizol (Life Technologies, Carlsbad, CA) and extracted RNA was treated with DNase I (Promega, Madison, WI). 2 μg of total RNA was reverse transcribed with MultiScribe TM MuLV reverse transcriptase (LifeTechnologies, Carlsbad, CA). 40 ng cDNA was used for quantitative polymerase chain reaction (PCR) amplification (Life Technologies, Carlsbad, CA) with 2X SYBR green PCR master mix (Life Technologies, Carlsbad, CA). Negative controls were included in samples where no reverse transcriptase was added for each RNA sample. The relative gene expression levels were normalized according to those of *Hprt1* and/or *18S* rRNA. Relative fold expressions were calculated using the comparative Ct method (Life Technologies, Carlsbad, CA). Standard deviations were calculated from the mean of the ΔCt values calculated from at least three independent RNA samples. Primers used are listed in S1 Table.

### shRNA

Cells were transfected with scrambled control or *Tcea3* mRNA specific shRNA constructs designed by the RNAi Consortium (TRC) in the pLKO.1 puro vector (Open Biosystems). Five constructs targeting *Tcea3* mRNA (shTcea3) were transfected using the TurboFect transfection reagent (Thermo Scientific) according to manufacturer’s protocol. Protein and RNA were harvested 48 hours post shRNA transfection or selected for stable cell lines with puromycin (2 μg/ml). Selected colonies were grown and validated for depletion.

### Western blot

Phosphate-buffered saline (PBS) was used to wash cells prior to lysing in RIPA buffer supplemented with protease inhibitors (Complete, Roche Diagnostics, Indianapolis, IN) and centrifugation was used to obtain clear lysates. Bradford’s assay (Bio-Rad, Hercules, CA) was used to determine protein concentration. 50 μg protein was loaded for each well of sodium dodecyl sulfate polyacrylamide gel electrophoresis (SDS-PAGE). Resolved proteins were then transferred onto a PVDF membrane using a tank blotter (Bio-Rad, Hercules, CA). Membranes were blocked using 5% milk in 1X Tris-buffered saline plus Tween 20 (TBST) and followed by incubation with primary antibody for overnight at 4°C. Later, TBST (1X) was used for washing membranes, then these membranes were incubated with the corresponding secondary antibody. After the incubation period, the blots were washed with 1X TBST and incubated with chemiluminescent substrate according to the manufacture’s protocol (SuperSignal, Pierce, Rockford, IL) and visualized by autoradiography. The antibodies used include anti-TCEA3 (T.160.5, Thermofisher Scientific), anti-MYOG (F5D, Developmental Studies Hybridoma Bank (DSHB)), anti-GAPDH (Millipore), anti-TUBULIN (E7, DSHB), anti-MYOD1 (5.8A, Santa Cruz Biotechnology (SCBT)), anti-RBP1 (A-10, SCBT) and anti-Myosin heavy chain (MF-20, DSHB). GAPDH / TUBULIN blots were used as loading controls except for fractionation assays where ACTIN was used as a loading control for both nuclear and cytoplasmic fractions.

### Cell transfections

Calcium phosphate transfections were performed according to standard protocols. TurboFect Transfection Reagent (Thermo Scientific) was used according to manufacturer’s protocol. For both, cells were allowed to grow 24 or 48 hours before harvesting RNA or protein for analysis or selected for stable cell lines.

### Chromatin immunoprecipitation (ChIP)

ChIP assays were performed and quantified as described previously [12] with the following modifications: 1x10^7^ cells were used for each immunoprecipitation and protein A agarose beads (Life Technologies, Carlsbad, CA) were used to immunoprecipitate the antibody:antigen complexes. Primers are described in S1 Table. The antibodies used for ChIP assays were anti-TCEA3 (T.160.5) (Thermofisher Scientific), anti-MYOD1 (5.8A, SCBT), anti-MYOG (F5D, DSHB) and anti-RPB1 (A-10, SCBT). Negative control immunoprecipitations utilized normal anti-mouse or anti-rabbit IgG (SCBT). The real time PCR was performed in triplicate. The results are represented as percentage of IP over input signal (% Input). All ChIP assays shown are representative of four independent experiments. Standard deviations (S.D.) was calculated and plotted as error bar.

### Co-immunoprecipitation (Co-IP)

Whole cell extracts were made in radioimmunoprecipitation assay (RIPA) buffer. 150–300 μg of extract was used for each immunoprecipitation. Extracts were incubated overnight with 1 μg of antibody at 4° C and antibody:antigen complexes were pulled down with protein A beads (Invitrogen). Antibodies used for immunoprecipitation included anti MYOG (F5D, DSHB), MYOD (5.8A, SCBT) and RNAPII (A10, Santa Cruz Biotechnologies). Western blot assays were used to check for the candidate interacting protein in each complex and the reciprocal factor was used to confirm the interaction. All immunoprecipitations were performed at least three times.

### Immunofluorescence

Cells were grown on cover slips, fixed with paraformaldehyde, blocked with 10% goat serum, 1.0% NP-40 in phosphate buffered saline (PBS) for one hour and washed with PBS. Primary antibodies against myosin heavy chain (MF20, DSHB) were incubated overnight at 4°C, washed with PBS and detected by Alexa Fluor-488 goat anti-mouse antibody (Life Technologies). Cell nuclei were stained by incubating with 1 μM DAPI (Life Technologies) for 5 min.

### Proliferation assay

4X10^4^ cells per well were seeded in 6-well plates and harvested on the indicated day for counting by hemocytometer. Cell viability was determined by using trypan blue staining. Cell counting was performed in duplicate and experiments were repeated twice.

### Luciferase assays

The Dual-Luciferase Reporter Assay System (Promega, Madison, WI) was used to assay for luciferase activity. 10T1/2 cells were seeded at a density of 5X10^3^ cells per well in 96-well plates and transfected with 0.4 μg of DNA. Transfections were normalized to Renilla luciferase. Transfections were performed in triplicate and all data sets were repeated at least three times.

### Statistics

Data are presented as means ± standard deviation (SD). Statistical comparisons were performed using unpaired two-tailed Student’s t tests, with a probability value of (p) < 0.05 taken to indicate significance.

## Results and discussion

### TCEA3 is regulated by myogenin

**Fig 1:**
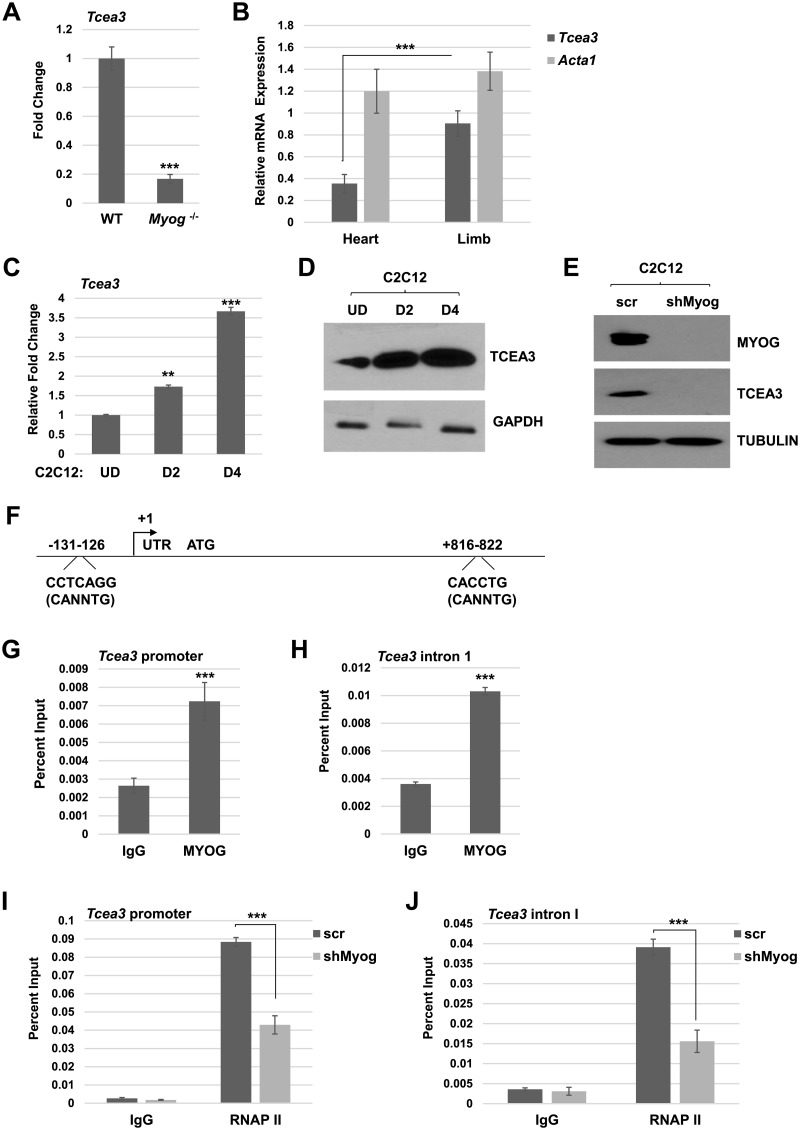
TCEA3 is regulated by myogenin. A *Tcea3* mRNA expression in E14.5 tongue tissue was quantified by qRT-PCR. B. *Tcea3* mRNA expression was quantified in 6W heart and hindlimb tissues by qRT-PCR. Data were normalized to the expression of *Hprt1*. C. TCEA3 expression increases upon differentiation. Time course analysis of *Tcea3* mRNA expression in C2C12 cells subjected to differentiation for the specified number of days (D) by qRT PCR. UD represents undifferentiated. D. Cells as in C. were assayed by western blot with indicated antibodies. E. C2C12 cell lines were transfected with shRNA constructs against *Myog* (shMyog) or the scrambled control (scr) and assayed by western blot analysis with indicated antibodies. F. Schematic of the *Tcea3* promoter. Transcription start site is labeled as +1. G—H. MYOG binds to the *Tcea3* promoter. ChIP assays were performed on C2C12 cells differentiated for 2 days with antibodies against myogenin and primers against E boxes present in the *Tcea3* promoter (-131 to -126) (G) and the first intron (+816 to +822) (H). I-J. Loss of MYOG inhibits RNAPII recruitment to the *Tcea3* promoter. ChIP assays were performed on cells as in E. with antibodies against RPB1, the largest subunit of RNAPII and primers against E boxes present in the *Tcea3* promoter (I) and first intron (J). All graphs represent three independent experiments. S.D. represents the error bars, **p<0.01, ***p<0.001.

In a microarray analysis on E14.5 murine tongue tissue from *myogenin*^*+/+*^ (WT) and *myogenin*^*-/-*^ (*Myog*^-/-^) embryos performed to identify the genetic program controlled by MYOG, *Tcea3* was identified as downregulated *in Myog*^*-/-*^ embryos [13]. Of the 140 genes found to be down regulated in *Myog*^*-/-*^ tongue tissue, *Tcea3* was the 58^th^ most down regulated gene in the array analysis [13]. This down regulation could be a direct or indirect effect of MYOG, but the result implied that *Tcea3* was regulated by MYOG and would be up regulated during differentiation. We confirmed the down regulation of *Tcea3* mRNA by qRT PCR in WT and *Myog*^*-/-*^ tongue tissue and found that *Tcea3* mRNA was down regulated in the absence of *myogenin* (Fig 1A). As *Tcea3* has been shown to be expressed in both heart and skeletal muscle [1], we sought to compare the relative expression in these tissues. Robust expression of *Tcea3* mRNA was found in the heart and hind limb skeletal muscle, with skeletal muscle expressing levels even higher than the heart (Fig 1B). We next confirmed the expression of *Tcea3* in C2C12 cells, an immortal murine cell line commonly used as a model for myogenesis. We found that *Tcea3* mRNA was expressed in C2C12 cells and, as anticipated, that *Tcea3* was upregulated upon differentiation at the level of mRNA (Fig 1C) and protein (Fig 1D).

### Myogenin directly regulates TCEA3

*Tcea3* was identified as downregulated upon the loss of *myogenin in vivo*. To confirm these results in C2C12 cells, we transiently depleted *myogenin* mRNA (shMyog) from C2C12 cells using shRNA constructs [12]. The depletion of MYOG confirmed at the protein level (Fig 1E). We found that with the depletion of MYOG, TCEA3 was also severely deregulated (Fig 1E). This result confirmed that MYOG directly or indirectly regulates TCEA3. To determine if MYOG directly activates *Tcea3*, we examined the *Tcea3* promoter for potential MRF binding sites. The MRFs bind to E-boxes with the consensus sequence of CANNTG and can also bind noncanonical binding sites [14, 15]. We found a noncanonical E-box immediately upstream of the *Tcea3* transcription start site that was identified by rVISTA as a MYOD1 binding site. In the first intron of *Tcea3*, rVISTA identified a consensus E-box (Fig 1F). To determine if MYOG bound to either of these potential binding sites, we performed ChIP assays for MYOG using primers to detect the potential binding elements in either in the promoter or the first intron. We found that MYOG binds to the *Tcea3* promoter and the first intron of *Tcea3*, showing that MYOG directly binds the *Tcea3* promoter (Fig 1G) and first-intron (Fig 1H). To confirm that myogenin was required for *Tcea3* mRNA expression, we performed ChIP assays for RNAPII in C2C12 cells depleted for myogenin (shMyog). We found that RNAPII recruitment to the *Tcea3* promoter and first intron were reduced when myogenin was depleted (Fig 1I and 1J).

### Depletion of TCEA3 downregulated muscle specific genes and impaired differentiation in C2C12 cells

**Fig 2:**
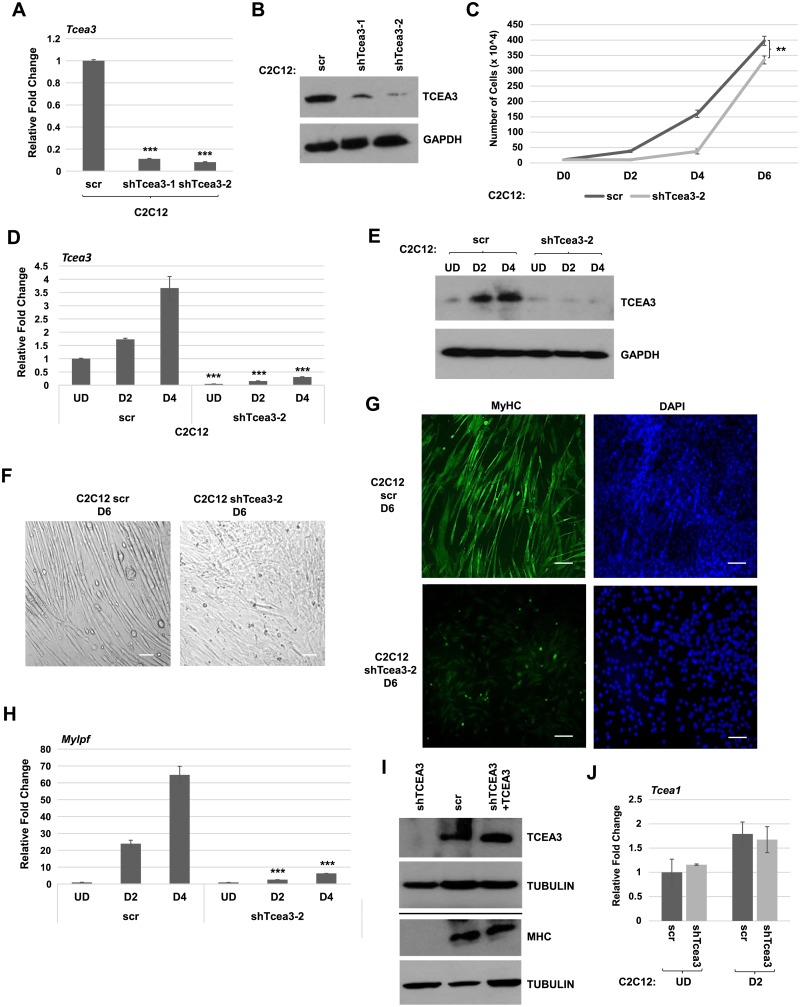
Depletion of TCEA3 impairs both proliferation and differentiation in C2C12 cells. A. Independent C2C12 cell lines expressing shRNA against *Tcea3* (shTcea3) and scrambled control (scr) were selected and analyzed by qRT-PCR. Data were normalized to the expression of *Hprt1*. B. Cells in A. were analyzed by western blot with the indicated antibodies. C. *Tcea3* depleted cells were assayed for proliferation. D-E. *Tcea3* depleted cells were differentiated for the indicated number of days and analyzed by qRT-PCR (D) and western blot assays (E).F. *Tcea3* depleted cells were differentiated for six days (D6). Images are bright field at 100X. G. Immunohistochemistry with antibodies against myosin heavy chain (MHC) was performed on cells as in F. DAPI was used to stain the nuclei. H. Gene expression of *Mylpf* mRNA in a time course of differentiation for *Tcea3* depleted cells and scr control. I. Restoration of TCEA3 restores differentiation in *Tcea3* depleted cells. *Tcea3* depleted cells were transfected with an expression construct for TCEA3 (pTCEA3), differentiated for two days and used for western blot analysis with the indicated antibodies. J. TCEA1 is not downregulated with the depletion of TCEA3. Cells in A. were analyzed by qRT-PCR for *Tcea1* mRNA at the indicated timepoints. S.D. represents the error bars, ***p<0.001.

To understand the role of TCEA3 in skeletal muscle, we depleted *Tcea3* in C2C12 cells using shRNA constructs (shTcea3). C2C12 cells were transfected with plasmids containing shTcea3, and stable clones were selected and screened to identify clones that were depleted for TCEA3. To first confirm that TCEA3 was depleted, we examined gene expression in independent clones of C2C12 cells transfected with shTcea3 and found that *Tcea3* mRNA was down regulated (Fig 2A). Next, we assayed for TCEA3 protein expression and found that TCEA3 protein was depleted as well (Fig 2B). As TCEA3 had been implicated in cell growth and differentiation in cancer cells, we asked if the depletion of TCEA3 affected proliferation. Cell growth analysis was performed and the growth curve showed that loss of TCEA3 decreased the proliferation rate of C2C12 cells (Fig 2C).

To determine how TCEA3 affected myogenic differentiation, the TCEA3 depletion lines were induced to differentiate. The results from both lines were very similar and only the results from one line are shown here. The depletion of TCEA3 was confirmed at the mRNA (Fig 2D) and protein level (Fig 2E) throughout the differentiation time course. We found that the cells did not appear to fuse and form myotubes normally by bright field microscopy (Fig 2F). To confirm this result, immunofluorescence using antibodies against myosin heavy chain, a marker for myogenic differentiation, was performed. No myotubes were observed in the TCEA3 depleted cells and myosin heavy chain positive cells were greatly reduced when compared to myosin heavy chain positive cells in cells transfected with scr vector control (Fig 2G). We also examined the expression of *myosin light chain*, *phosphorylatable*, *fast skeletal muscle (Mylpf)* mRNA and found that it was highly downregulated in the absence of TCEA3 (Fig 2H). To confirm that these effects were the result of the absence of TCEA3, we rescued the expression of TCEA3 in the C2C12 cells depleted for TCEA3 using a TCEA3 expression construct. We found that expression of TCEA3 restored TCEA3 expression and rescued the expression of MyHC (Fig 2I). Skeletal muscle cells express both TCEA3 and TCEA1, and our results indicate that TCEA1 cannot compensate for the loss of TCEA3. It was also possible that loss of TCEA3 would repress *Tcea1*. To test this, we assayed for the expression of *Tcea1* mRNA in TCEA3 depleted cells and found that *Tcea1* mRNA was not downregulated in the absence of TCEA3 (Fig 2J), suggesting that TCEA1 is present, but cannot compensate for the loss of TCEA3. Together, these data indicate that TCEA3 is required for normal cell proliferation and skeletal muscle differentiation.

### TCEA3 promotes muscle specific gene expression

**Fig 3:**
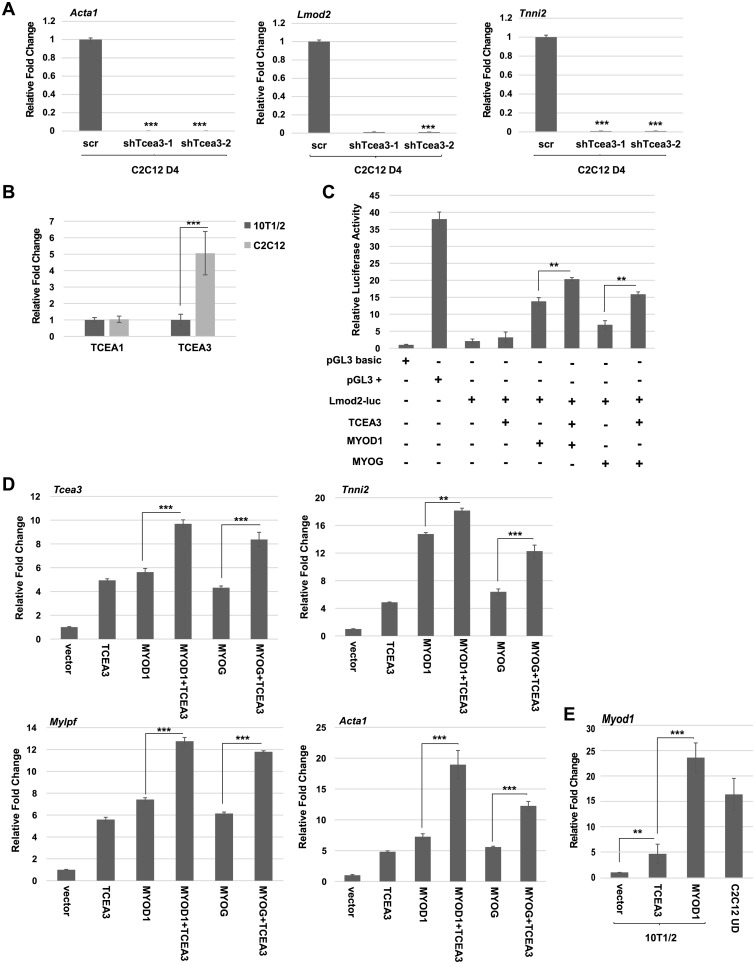
TCEA3 induces MRF activity. A. *Tcea3* depleted cells (shTcea3) and scr control were differentiated for four days and analyzed for MRF dependent gene expression by qRT-PCR. B. TCEA3 is not highly expressed in 10T1/2 cells. mRNA expression of TCEA1 and TCEA3 were assayed by qRT-PCR in 10T1/12 cells and C2C12 cells (UD). C. TCEA3 induces the activity of MYOG and MYOD1 on muscle-specific luciferase reporter constructs. 10T1/2 cells were transiently transfected with the indicated constructs. Values are represented with respect to a luciferase vector with no promoter (pGL3 basic). pGL3(+) represents a luciferase vector with an SV40 promoter. *Lmod2*-luc is a MRF dependent luciferase vector. D. TCEA3 enhances endogenous MRF target gene expression. 10T1/2 cells were transfected with expression constructs for MYOG, MYOD and TCEA3 as indicated and gene expression was determined by qRT-PCR. Data were normalized to the expression of *Hprt1*. E. TCEA3 modestly activates MYOD1. Cells in D. and undifferentiated C2C12 cells (UD) were assayed for MYOD1 mRNA by qRT-PCR. S.D. represents the error bars, **p<0.01 ***p<0.001.

We next asked if additional MRF dependent gene expression was dependent on TCEA3. Levels of *actin 1 (Acta1)*, *leiomodin 2 (Lmod2)* and *troponin I*, *skeletal*, *fast 2 (Tnni2)* mRNA were examined by qRT-PCR in C2C12 cells depleted for TCEA3 and we found that all three genes were highly down regulated (Fig 3A). 10T1/2 cells, a fibroblast cell line that can be induced to express muscle specific genes upon expression of the MRFs[16] were used for this assay. The expression of TCEA3 had not been examined in these cells, so we first sought to determine if TCEA3 was expressed in these cells. mRNA expression for TCEA1 and TCEA3 was examined in 10T1/2 cells and C2C12 cells, and we found that while C2C12 and 10T1/2 cells contained roughly equivalent levels of TCEA1 mRNA, C2C12 cells, even in the undifferentiated state, contained significantly higher levels of TCEA3 (Fig 3B). To understand how TCEA3 impacted gene activation mediated by MYOG and MYOD1, a muscle specific luciferase reporter was used. The promoter construct used had been previously characterized as highly dependent on MYOG *in vivo* and contained a promoter element of murine *leiomodin 2* (*Lmod2*; base pairs −10 to −458) [13]. 10T1/2 cells were transfected with the *Lmod2* luciferase reporter and expression constructs for MYOG, MYOD1 and TCEA3. We found that transfection with TCEA3 enhanced activation of the *Lmod2* luciferase reporter and TCEA3 also acted as an inducer of activation by MYOG and MYOD1 (Fig 3C).

To determine if TCEA3 could promote endogenous muscle specific gene expression, we assayed for the effects of TCEA3 on muscle-specific genes in 10T1/2 cells, which do not normally express these genes. 10T1/2 cells were transfected with MYOG, MYOD1, and TCEA3 individually, and in combination. Genes chosen for analysis were *Lmod2*, *Tnni2*, *Mylpf* and *Acta1*. Gene expression of each of these genes is dependent on the MRFs. It is noteworthy that *Tcea3* was upregulated itself upon transfection with MYOD1 or MYOG, confirming the regulation of TCEA3 by the MRFs (Fig 3D). We found that transfection of TCEA3 modestly induced muscle-specific gene activation and also acted as a co-activator for both MYOG or MYOD1 (Fig 3D). The modest activation of muscle specific genes by TCEA3 in the absence of the MRFs was intriguing, as it suggested that TCEA3 might have an MRF independent role in gene activation. It was also possible that TCEA3 might promote MRF gene activation. To test this, we examined the expression of MYOD1 in 10T1/2 cells transfected with expression constructs for TCEA3 or MYOD1. mRNA expression was compared to the mRNA expression normally seen in undifferentiated C2C12 cells. We found that TCEA3 does modestly induce MYOD1 expression, but not to the levels seen upon MYOD1 expression or normally seen in C2C12 cells (Fig 3E). The modest activation of MYOD1 is consistent with the modest activation of muscle specific genes seen with the sole expression of TCEA3, suggesting that the activation is through the MRFs and that TCEA3 acts in a feed forward loop to promote expression of the MRFs which regulate its own transcription. Taken together, these results support that TCEA3 functions as a co-factor of MRF driven gene expression.

### TCEA3 interacts with MYOG and MYOD1

**Fig 4:**
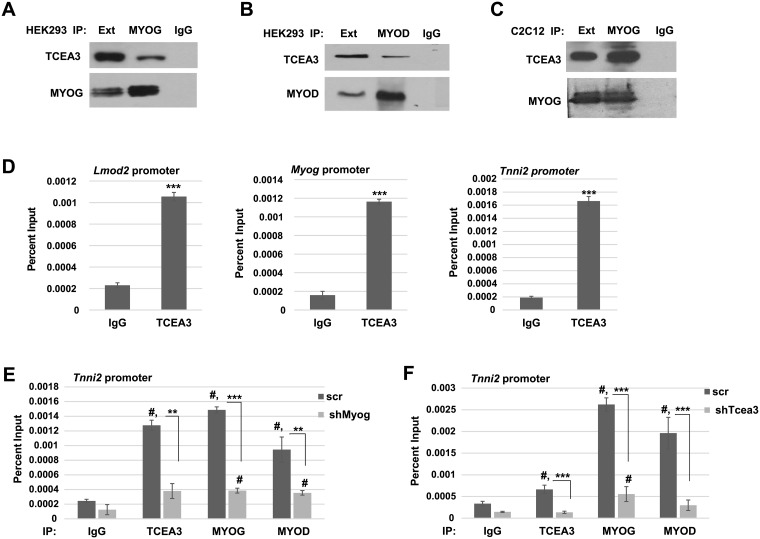
TCEA3 interacts with MYOG and MYOD1. A-B. Expression constructs for TCEA3 and MYOG or MYOD1 were co-transfected into HEK293 cells and immunoprecipitated with antibodies against MYOG (A) or MYOD1 (B). Immunoprecipitation was detected with antibodies against TCEA3. Cell extract is labeled Ext. C. Differentiated C2C12 cell extract (D4) was immunoprecipitated with antibodies against MYOG and detected with antibodies against TCEA3 and MYOG. D. TCEA3 binds to MRF dependent genes. ChIP assays were performed with antibodies against TCEA3 and primers to MRF dependent promoters as indicated. E. Myogenin recruits TCEA3 to promoters. ChIP assays were performed on C2C12 cells depleted for *Myog* (shMyog) and scr control with antibodies against TCEA3, MYOG and MYOD1. S.D. represents error bars and #p <0.05 (vs IgG) **p<0.01, ***p<0.001 as indicated. F. TCEA3 depletion decreases the binding of myogenin on a muscle-specific gene promoter. ChIP assays were performed in C2C12 cells depleted for *Tcea3* (shTCEA3) and scr control with antibodies against TCEA3, MYOG and MYOD1. S.D. represents error bars and #p <0.05 (vs IgG) **p<0.01, ***p<0.001 as indicated.

Our results showed that TCEA3 acted as a co-activator for MYOD1 and MYOG. To understand the basis for this effect, we asked if TCEA3 could interact with MYOD1 or MYOG using co-immunoprecipitation assays (co-IP). We expressed both exogenous TCEA3 and MYOG in HEK293 cells by transfection with expression constructs for both proteins. Whole cell lysates were immunoprecipitated with antibodies against MYOG, the associated complex was isolated and used for western blot assays with antibodies against TCEA3 to assay for the interaction. We found that TCEA3 was immunoprecipitated with MYOG (Fig 4A). Antibodies against TCEA3 were used to confirm the immunoprecipitation (Fig 4A). Because our results had shown that TCEA3 promoted the activity of MYOD1 and MYOG, we also if TCEA3 interacted with MYOD1. The co-IP was repeated with expression constructs for TCEA3 and MYOD1, using antibodies against MYOD1 for the immunoprecipitation and we found that TCEA3 also interacted with MYOD1, suggesting that TCEA3 interacts with both MRFs (Fig 4B). To confirm that the interaction could be observed with endogenous proteins, we repeated the co-immunoprecipitation in differentiated C2C12 cells. Whole cell lysates were immunoprecipitated with antibodies against MYOG and the associated complex was used for western blot assays with antibodies against TCEA3. We found that endogenous TCEA3 was immunoprecipitated with MYOG in C2C12 cells (Fig 4C).

### TCEA3 recruitment to promoters is dependent on myogenin

We had found that TCEA3 both interacted with the MRFs and promoted MRF dependent gene activation. To understand the mechanism by which TCEA3 promoted MRF dependent gene activity, we asked if TCEA3 bound to MRF dependent gene promoters. *Myogenin (Myog)*, which is regulated by MYOD1, was used as an MRF dependent promoter in this assay. We also examined the enrichment of TCEA3 to the *Lmod2* and *Tnni2* promoters, as we had shown that these genes were downregulated upon the depletion of TCEA3. ChIP assays were used to assay for the binding of TCEA3 and we found that TCEA3 was bound to the promoters of *Myog*, *Lmod2* and *Tnni2* (Fig 4D).

Our results showed that TCEA3 both interacted with MYOG and was recruited to MYOG dependent genes. Thus, we asked if the recruitment of TCEA3 to muscle-specific genes was dependent on MYOG. ChIP assays for TCEA3, MYOD1 and MYOG were performed on C2C12 cell lines depleted for MYOG. We found that, as anticipated, depletion of MYOG reduced the binding of both MYOD1 and MYOG to the *Tnni2* promoter (Fig 4E). We also found that the promoter binding of TCEA3 was significantly decreased upon the depletion of MYOG (Fig 4E). These data suggest that MYOG is required for TCEA3 binding to the promoter of *Tnni2*.

To understand if this recruitment was reciprocal, we also asked if TCEA3 promoted the recruitment of MYOD1 and MYOG to the *Tnni2* promoter by ChIP assays. Reduced recruitment of MYOD1 and MYOG on the *Tnni2* promoter was observed in TCEA3 depleted cells (Fig 4F). The results show that TCEA3 cooperates with the MRFs to regulate muscle specific gene expression.

### TCEA3 binds to RNAPII and travels with RNAPII during transcript elongation

**Fig 5:**
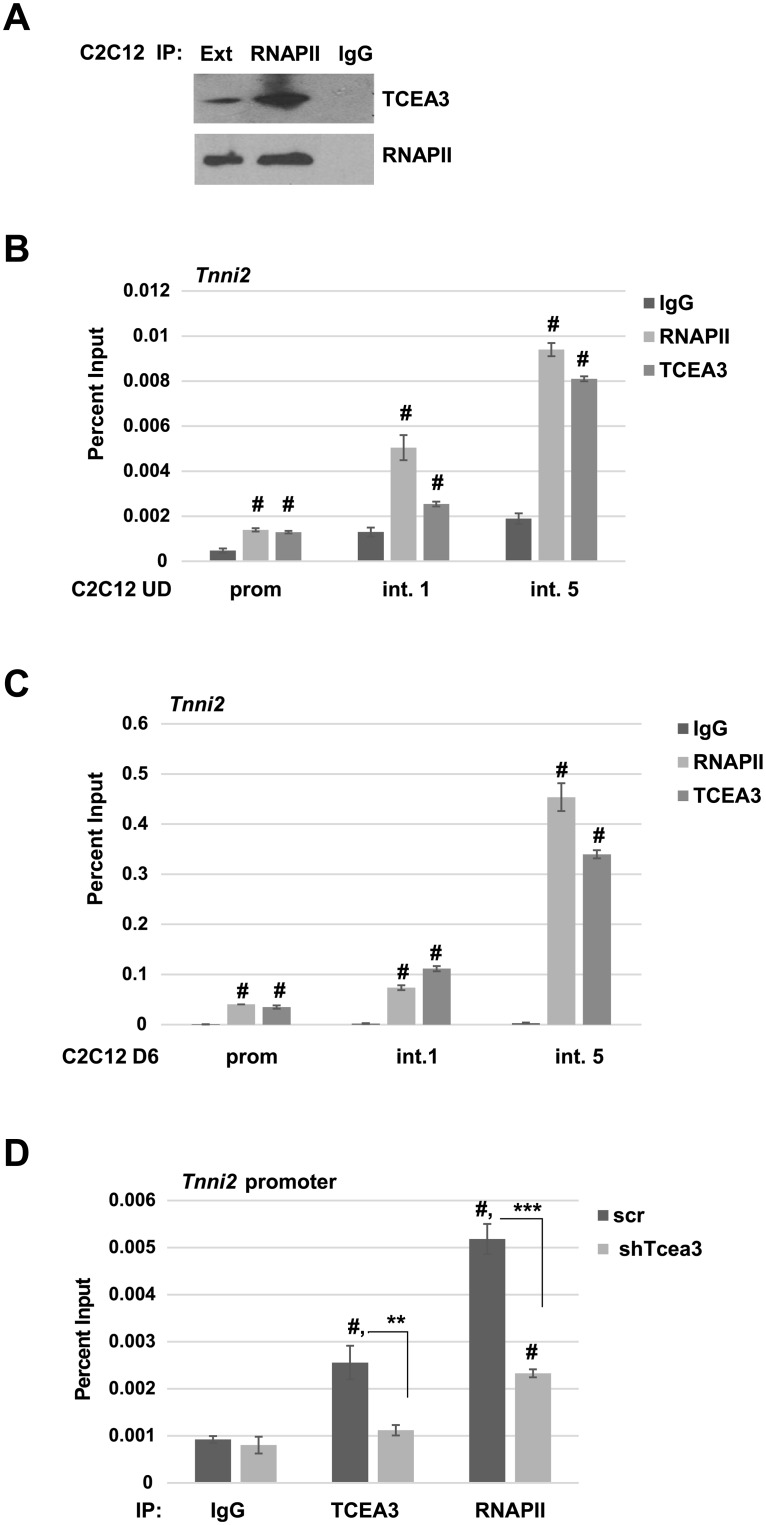
TCEA3 binds to RNAPII, promotes the recruitment of RNAPII and travels with elongating RNAPII. A. TCEA3 binds to RNAPII. Cell extracts from C2C12 cells differentiated for 2 days (D2) were immunoprecipitated with antibodies against RPB1, the largest subunit of RNAPII. The western blot was probed with antibodies against TCEA3 and RPB1. Cell extract is labelled Ext. B. ChIP assays were performed on proliferating C2C12 cells (UD) with antibodies against TCEA3 and RNAPII and detected with primers corresponding to the promoter, intron-1 and intron-5 of *Tnni2*. S.D. represents error bars and #p <0.05 (vs IgG). C. ChIP assays were performed as in B. on C2C12 cells following differentiation for 6 days (D6). D. TCEA3 promotes the recruitment of RNAPII. ChIP assays were on C2C12 cells depleted for *Tcea3* or scr control following 2 days of differentiation (D2) with antibodies against TCEA3 and RNAPII and primers against the *Tnni2* promoter. S.D. represents error bars **#**p<0.05(vs IgG) **p<0.01, ***p<0.001 as indicated.

As TCEA3 is a member of the TFIIS family, we asked if TCEA3 binds to RNAPII as does TFIIS. TCEA3 has been shown to promote transcript cleavage by RNAPII [1], strongly suggesting that TCEA3 does interact with RNAPII. However, TCEA3 is most divergent in the linker region of TFIIS, which has been the region of TFIIS shown to be required for the interaction [17]. RNAPII was immunoprecipitated from C2C12 cells and we found that TCEA3 was immunoprecipitated as well (Fig 5A).

Next, we asked if TCEA3 travelled with RNAPII during elongation by ChIP assays. We found that TCEA3 co-localized with RNAPII and could be found at the promoter and throughout the coding region of *Tnni2*. This recruitment could be observed in proliferating C2C12 cells, when *Tnni2* was not highly expressed (Fig 5B). However, the recruitment of both RNAPII and TCEA3 was greatly enhanced in C2C12 cells after six days of differentiation, when *Tnni2* is highly expressed (Fig 5C). Thus, the recruitment of TCEA3 positively correlated with the enrichment of RNAPII and the transcription activity of the gene.

Finally, we asked if TCEA3 affected the recruitment of RNAPII to MRF dependent promoters. ChIP assays were performed in C2C12 cells depleted for TCEA3. We found that the recruitment of RNAPII was inhibited in the absence of TCEA3 (Fig 5D). As anticipated, the recruitment of TCEA3 was also reduced upon TCEA3 depletion. Our data suggest that TCEA3 promotes RNAPII recruitment to the promoters of muscle specific genes and that TCEA3 travels with the elongating RNAPII to promote productive elongation.

### TCEA3 translocates to the nucleus upon differentiation

**Fig 6:**
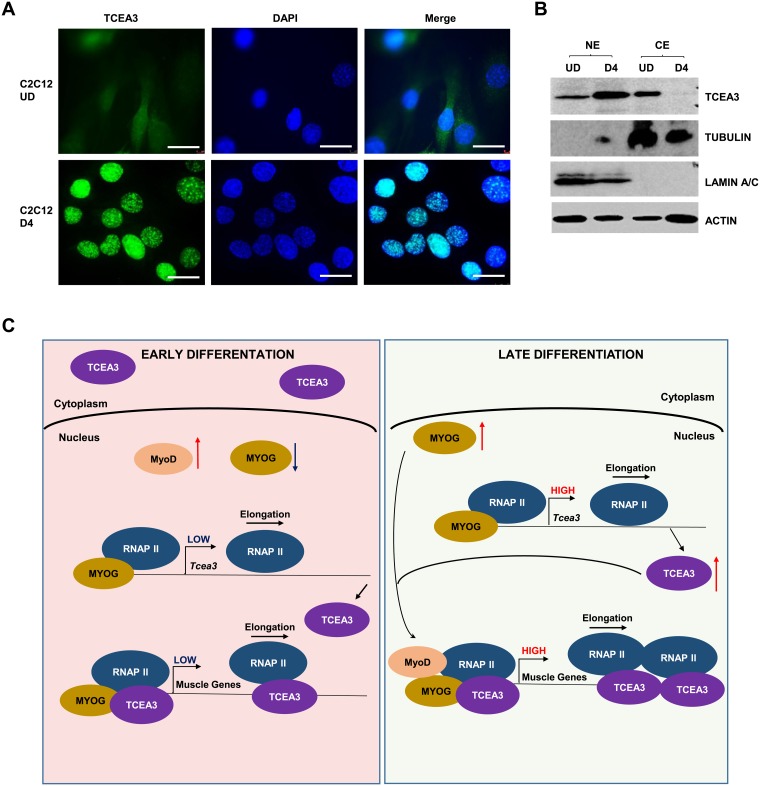
TCEA3 translocates to the nucleus upon differentiation. A. TCEA3 localization in C2C12 cells during proliferation (UD) and after 4 days of differentiation (D4). Immunofluorescence was performed with antibodies against TCEA3. Nuclei were stained with DAPI. Images were taken at 400x magnification. Scale bars represent 25 μm. B. Both nuclear (NE) and cytoplasmic (CE) fractions of proteins were extracted from proliferating (UD) and differentiated (D6) C2C12 cells and probed for TCEA3, LAMIN A/C (loading control for nucleus), TUBULIN (loading control for cytoplasm) and ACTIN. C. Model for the function of TCEA3 in myogenesis. During early differentiation, TCEA3 levels are low and TCEA3 is present in the nucleus and cytoplasm. Upon differentiation, MYOG levels increase and induce *Tcea3*, which translocates to the nucleus and enhances MRF driven gene expression by promoting RNAPII recruitment and elongation.

TCEA3 has recently been shown to promote differentiation and myotube fusion [9], consistent with the results shown here. In this previous study, TCEA3 was shown to be localized primarily in the cytoplasm [9]. This was surprising to us as our results strongly suggested a nuclear function. To address this question, we examined the cellular localization of TCEA3 in both proliferating C2C12 cells and in differentiated C2C12 cells by immunofluorescence. In proliferating cells, we saw that TCEA3 was present at low levels in both the cytoplasm and nucleus (Fig 6A). However, upon differentiation, TCEA3 underwent an upregulation in expression and a dramatic cytoplasmic to nuclear translocation which left TCEA3 undetectable in the cytoplasm (Fig 6A).

To confirm this result, we also fractionated cytoplasmic and nuclear extracts in C2C12 cells before and after differentiation and probed the extracts for TCEA3 and markers to confirm the fractionation. We observed a decrease in cytoplasmic TCEA3 and an increase of nuclear TCEA3 upon differentiation (Fig 6B).

In summary, our results show that the level of TCEA3 expression increases during differentiation and that TCEA3 translocates to the nucleus upon differentiation; further supporting its role as a transcription elongation factor which induces muscle differentiation in normal skeletal muscle cells (Fig 6C).

In this work, we show that TCEA3 enhances skeletal muscle differentiation by promoting MRF dependent gene expression. TCEA3 had previously been shown to promote myogenesis in bovine satellite cells, but was shown to be localized to the cytoplasm [9]. This work has recently been expanded to C2C12 cells showing that TCEA3 is localized to the cytoplasm and promotes differentiation [18]. This localization was difficult to rectify with the identification of TCEA3 as a member of the TFIIS transcription elongation factor family. Here, we show that TCEA3 is localized to the cytoplasm and nucleus in proliferating cells, but translocates to the nucleus upon differentiation. This finding reconciles our work with the previously reported studies and supports a common function in vertebrates.

TCEA3 has been shown to interact with cytoplasmic annexin A1 [18], which is also known to promote differentiation [19]. Annexin A1 regulates TGF-ß signaling [20] and TCEA3 was shown to regulate the TGF-ß pathway through annexin A1 [18]. We show that TCEA3 depleted cells have a reduced proliferation rate, and the interaction with annexin A1 and the TGF-ß pathway may underlie this defect. TCEA3 is present in the cytoplasm during proliferation and the interaction with annexin A1 and the proliferation defect strongly suggests that TCEA3 functions in both the cytoplasm and nucleus to regulate both the proliferation and differentiation of skeletal muscle cells.

## Conclusions

We found that TCEA3 is directly regulated by myogenin, which upregulates TCEA3 upon differentiation. TCEA3 is then recruited to MRF dependent genes through interactions with MYOD1, MYOG and RNAPII and enhances RNAPII recruitment to promoters. TCEA3 then travels with RNAPII along the coding region of the gene. Thus, TCEA3 is a previously undescribed co-factor in MRF dependent gene activation. Our data shows that TCEA3 both contributes to RNAPII recruitment to promoters as well as travels with RNAPII during elongation, suggesting that TCEA3 contributes to enhancing elongation by RNAPII. Recombinant *Xenopus* proteins representing the three TFIIS family members have been shown to cleave RNA in a stalled RNAP II elongation complex [1], suggesting that all three family members can function as transcription elongation factors. It is intriguing that skeletal muscle expresses TCEA3, which is the most divergent from the other two TFIIS family members in the linker region that was the region shown to interact with RNAPII [17]. Given this difference, we anticipated that TCEA3 might not interact with RNAPII, but our data clearly show that TCEA3 can interact with RNAPII. It is possible that the longer linker region in TCEA3 allows for additional interactions that promote its activity, such as the interactions with MYOG and MYOD1. TCEA3 has also been shown to be a target of P53, where TCEA3 then contributes to activation of selective P53 targets such *Bax*, but not *Cdkn1a* (p21) [21]. These interactions may also function in skeletal muscle, but it is intriguing that our results suggest a similar regulatory circuit, with MYOG activating TCEA3 which then promotes MYOG activation of gene targets.

We also see that TCEA3 can modestly promote the activation of MYOD1, which activates MYOG. Thus, TCEA3 reinforces its own expression through this feed forward loop. Our results also suggest that maintaining productive elongation by RNAPII is a critical function in myogenesis. While genome wide gene expression was not assayed, every MYOG dependent gene examined in this study was severely down regulated in the absence of TCEA3, suggesting that the ability to overcome transcriptional arrest sites is required for many muscle specific gene coding units. Skeletal muscle cells also express the ubiquitous TCEA1, but clearly TCEA3 seems to have unique function as a MYOG dependent co-factor for the MRFs in promoting skeletal muscle differentiation that cannot be compensated for by additional transcription elongation factors such as TCEA1. The translocation of TCEA3 upon differentiation is intriguing and suggests that this an additional mechanism to prevent precocious activation of the MRFs and assure precise control of myogenic differentiation.

## Supporting information

S1 Table Oligonucleotides used in study. (DOCX)
