# Supplementary material for: The transcription elongation factor TCEA3 promotes the activity of the myogenic regulatory factors
Source: PLoS One. 2019 Jun 3;14(6):e0217680. doi: 10.1371/journal.pone.0217680 (PMC6546274; doi:10.1371/journal.pone.0217680)
Supplement: S1 Table — (DOCX) [file pone.0217680.s001.docx]

**S1 Table**

**Oligonucleotides used in study**

qRT-PCR:

18S rRNA F 5’ CGCCGCTAGAGGTGAAATTCT 3’

R 5’ CGAACCTCCGACTTTCGTTCT 3’

HPRT1 F 5’ TGACACTGGCAAAACAATGCA 3’

R 5’ GGTCCTTTTCACCAGCAAGCT 3’

MyoD F 5’ GCCGGTGTGCATTCCAA 3’

R 5’ CACTCCGGAACCCCAACAG 3’

Myogenin F 5’ GACCTGATGGAGCTGTATGAG 3’

R 5’ CTGAAGGTGGACAGGAAGG 3’

Tnni2 F 5’ GCCGCCGAGAATCTGAGA 3’

R 5’ GACATGGAGCCTGGGATGTG 3’

Tcea3 F 5’ GCTGAACAGTTGCCAGATGTC 3’

R 5’ GCAGCCGCTTCCAGTTTTTAAT 3’

Tcea1 F 5’ GAATGACAGCAGAGGAAATGG 3’

R 5’ CATTGGTTCTTCAGCACTACG 3’

Chromatin Immunoprecipitation (ChIP) assay:

Tnni2 int 5 F 5’ GCAGAGGGTAAACTGAGGC 3’

R 5’ CCATGCCAGACTTCTCCTC 3’

Tnni2 int1 F 5’ CTATCTCCCTGCCCTTTGTG 3’

R 5’ CACCTCCTCACTGTCCCAG 3’

Tnni2 prom F 5’CTCCATGCCTAGCCTCAC 3’

R 5’GGTGAGGGACTGCAGTACG 3’

Tcea3 E1 F 5’ GCAAACACACCGGACACTC 3’

R 5’ GAACCCTCATGCTCGAATCT 3’

Tcea3 E2 F 5’ GACTGAGGGCTCAGGGAC 3’

R 5’ CTGAAGTGCCAGGAGTTCTG 3’

IgH F 5’ GCCGATCAGAACCAGAACACCTGC 3’

R 5’ TGGTGGGGCTGGACAGAGTGTTTC 3’

Myog E1,2 F 5’ CTCCCCCACCTGACATTCTA 3’

R 5’ GGCTGGCCTCTTCTATTCCT 3’
